# Profile of gut microbiota and serum metabolites associated with metabolic syndrome in a remote island most afflicted by obesity in Japan

**DOI:** 10.1038/s41598-022-21708-0

**Published:** 2022-10-14

**Authors:** Tsugumi Uema, Jasmine F. Millman, Shiki Okamoto, Takehiro Nakamura, Kiyoto Yamashiro, Moriyuki Uehara, Ken-ichiro Honma, Minoru Miyazato, Asuka Ashikari, Seiichi Saito, Shiro Maeda, Minako Imamura, Hajime Ishida, Masayuki Matsushita, Koshi Nakamura, Hiroaki Masuzaki

**Affiliations:** 1 Division of Endocrinology, Diabetes and Metabolism, Hematology, Rheumatology, (Second Department of Internal Medicine), Graduate School of Medicine, University of the Ryukyus, 207 Uehara, Nishihara, Okinawa 903-0215 Japan; 2 Department of Systems Physiology, Graduate School of Medicine, University of the Ryukyus, Uehara, Nishihara, Okinawa Japan; 3 Department of Urology, Graduate School of Medicine, University of the Ryukyus, Uehara, Nishihara, Okinawa Japan; 4 Department of Advanced Genomic and Laboratory Medicine, Graduate School of Medicine, University of the Ryukyus, Uehara, Nishihara, Okinawa Japan; 5 Division of Clinical Laboratory and Blood Transfusion, University of the Ryukyus Hospital, Uehara, Nishihara, Okinawa Japan; 6 Department of Human Biology and Anatomy, Graduate School of Medicine, University of the Ryukyus, Uehara, Nishihara, Okinawa Japan; 7 Department of Molecular and Cellular Physiology, Graduate School of Medicine, University of the Ryukyus, Uehara, Nishihara, Okinawa Japan; 8 Department of Public Health and Hygiene, Graduate School of Medicine, University of the Ryukyus, Uehara, Nishihara, Okinawa Japan

**Keywords:** Clinical microbiology, Endocrinology

## Abstract

Numerous studies have revealed distinct differences in the profiles of gut microbiota between non-obese and obese individuals. To date, however, little is known if any disparities in the community of gut microbiota exist between metabolically healthy obese (MHO) and metabolically unhealthy obese (MUO) subjects. We therefore aimed to comprehensively characterize the gut microbiota and circulating metabolites in serum from both MHO and MUO residing in the remote island, Kumejima, where the prevalence of obesity is one of the highest in Japan, and explored possible correlations between the gut microbiota profile and markers of metabolic syndrome. Results revealed that MUO showed significantly higher levels of genera such as *g_Succinivibrio, g_Granulicatella, g_Brachyspira, g_Oribacterium* and *g_Atopobium* in comparison to MHO. Moreover, abundance of *g_Succinivibrio, g_Brachyspira* and *g_Atopobium* were positively correlated with value of fasting insulin, HOMA-R, circulating triglycerides, diastolic blood pressure, BMI, body weight, waist circumference and HbA1c. In addition, MUO compared to MHO showed an imbalance of serum metabolites, with a significant elevation in 2-oxoisovaleric acid, pyruvic acid, 2-hydroxybutyric acid, and creatine. Our data highlight unmet needs in precision approaches for the treatment of obesity, targeting the gut microbiota profile and serum metabolites in a distinct population affected by obesity.

## Introduction

Globally, obesity has reached to pandemic levels and represents a major risk for non-communicable diseases such as cardiovascular diseases and type 2 diabetes mellitus (T2DM); comprising greater than 70% of all deaths world-wide^1^. Obesity is generally characterized by a body mass index(BMI) ≥ 30 kg/m^2^ by the World Health Organization (WHO). On the other hand, obesity in Japan is classified as having a BMI ≥ 25 kg/m^2^, based on the observation that east and south-east Asians including Japanese represent a considerable risk elevation in obesity-related comorbidities at a much lower BMI than Caucasians, Hispanics and African people^2^.

The most southern prefecture, in Japan, Okinawa, comprises of a number of smaller islands, consisting of a distinctly different lifestyle and culture to that of the mainland of Japan, and was once world-renowned as being home to a large proportion of especially healthy, long-living individuals. However, Okinawa currently shows one of the highest prevalence of obesity and T2DM in Japan under the expeditious adoption of western style diets and sedentary lifestyle^3^. Among a variety of islands in Okinawa, particularly Kumejima Island has limited food access and availability, in turn creating a highly-obesogenic environment by limiting food choices to unhealthy and high calorie food items.

Cumulative evidence is emerging regarding the association between obesity and the profile of gut microbiota^4,5^, with lean individuals showing distinctly different profile of gut microbiota compared to those who are obese^6^. Both animal and human studies have demonstrated that a significant decrease in microbial diversity and also an imbalance in metabolomic and fermentation-related markers in states of obesity and T2DM^7,8^. Importantly, an elevated Firmicutes to Bacteroidetes ratio appears to be representative in animal models of obesity, however, this finding is not consistent with human studies^4^. In contrast, in humans, certain gut microbiota such as *Oscillospira, Lactobacillus* and *Alistipes*^6^ as well members of the Clostridia class which are associated with reduction of systemic inflammation are characteristically depleted in obese people as compared to lean subjects^9^.

Animal studies and a number of human studies have also revealed a valuable role of certain microbial metabolites such as short chain fatty acids (SCFAs), produced by fermentation of fiber, in the prevention and improvement of obesity and its comorbidities^10^. Conversely, a series of serum metabolites involved in glucose and lipid dysmetabolism such as pyruvic acid and hydroxybutyrate are elevated in obese and T2DM humans and animals^11,12^.

Although evidence appears compelling regarding the detrimental effects of obesity on the profile of gut microbiota, there is a controversy regarding the relationship between metabolic risk and obesity per se. Distinctively, as opposed to metabolically unhealthy obese (MUO), metabolically healthy obese (MHO) individuals show relatively lower levels of visceral fat accumulation^13^, are more sensitive to insulin and exemplify less inflammatory status^14,15^. However, a large gap between basic and clinical research still remains regarding the gut microbiota and circulating metabolites implicated in MUO.

**Figure 1:**
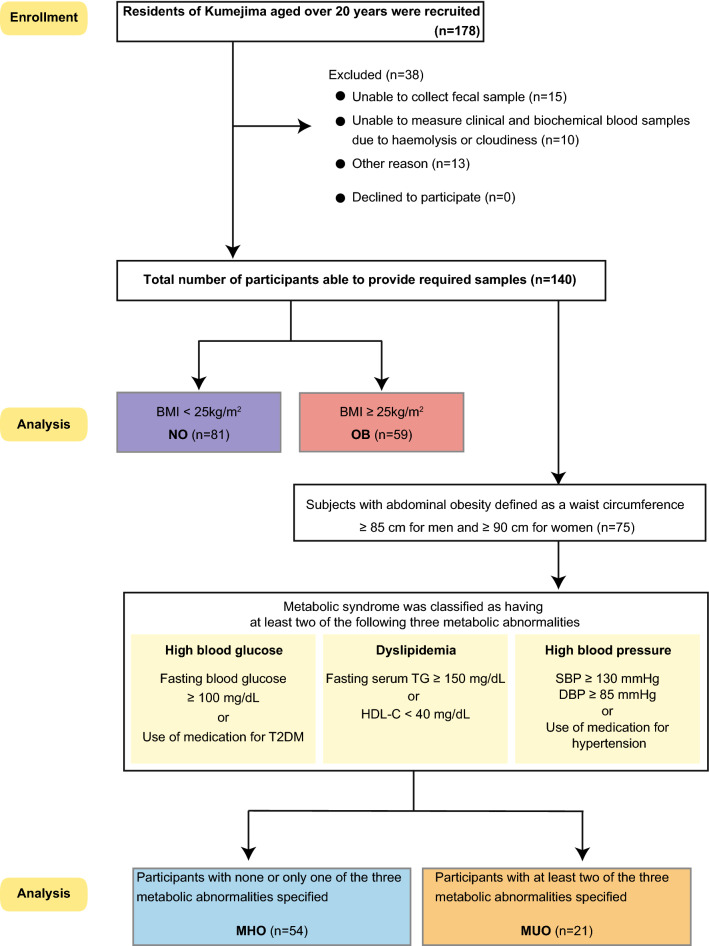
Study scheme. NO; non-obese (n = 81), OB; obese, (n = 59), MHO; metabolically healthy obese (n = 54) and MUO; metabolically unhealthy obese (n = 21). Graphics were made using the Adobe Illustrator (version: 5.8.0.592).

In this context, the present study aimed to characterize the profile of gut microbiota and serum metabolites in non-obese (NO) and obese (OB) subjects, with a special focus on MHO and MUO inhabitants of the remote island, Kumejima, where the prevalence of obesity is one of the highest in Japan (Fig. 1).

## Results

### Subject characteristics

A number of significant differences in anthropometric, clinical and biochemical parameters were observed between NO (n = 81) and OB (n = 59) (Table 1). Body weight, BMI and waist circumference were significantly higher in OB compared to NO (NO 56.89 ± 1.05, OB 71.66 ± 1.23, NO 22.42 ± 0.26, OB 27.93 ± 0.3 and NO 83.78 ± 0.81, OB 95.63 ± 0.95, *p* < 0.0001, respectively). NO and OB subjects also showed differences in a systolic blood pressure (SBP) (NO 128.89 ± 2.03, OB 138.31 ± 2.38, *p* = 0.0031) and a diastolic blood pressure (DBP) (NO 75.33 ± 1.25, OB 81.27 ± 1.46, *p* = 0.0024), with these parameters being elevated in the OB. Regarding biochemical parameters, compared to NO, the OB showed significantly higher levels of total-cholesterol (TC) (NO 198.33 ± 4.03, OB 215.39 ± 4.72, *p* = 0.0068), high-density lipoprotein-cholesterol (HDL-C) (NO 63.35 ± 1.78, OB 56.44 ± 2.09, *p* = 0.013), low-density lipoprotein-cholesterol (LDL-C) (NO 114.4 ± 3.46, OB 131.54 ± 4.05, *p* = 0.0016) and triglyceride (TG) (NO 81 (59–123), OB 134 (91–165), *p* < 0.0001), respectively. OB as opposed to NO subjects also had higher hemoglobin A1c (HbA1c) (NO 5.5 (5.3–5.7), OB 5.6 (5.4–6), *p* = 0.015), glucose (NO 81 (75.5–86), OB 83 (79–90), *p* = 0.047), insulin (NO 3.6 (2.9–5), OB 4.9 (3.5–8.5), *p* = 0.001) and Homeostatic Model Assessment of Insulin Resistance (HOMA-R) (NO 0.77 (0.55–1.005), OB 1.09 (0.66–1.8), *p* = 0.0007), respectively.

**Table 1:** Characteristics of NO and OB.

Anthropometric and clinical parameters	NO		OB		*p* value
	Mean	SEM	Mean	SEM	
n (M:F)	81 (37:44)		59 (33:26)		
Age (years)	56	1.47	56	1.72	0.96
Height (cm)	159.02	1.04	159.92	1.22	0.58
Body weight (kg)	56.89	1.05	71.66	1.23	< 0.0001
BMI (kg/m^2^)	22.42	0.26	27.93	0.3	< 0.0001
Waist circumference (cm)	83.78	0.81	95.63	0.95	< 0.0001
Systolic blood pressure (mmHg)	128.89	2.03	138.31	2.38	0.0031
Diastolic blood pressure (mmHg)	75.33	1.25	81.27	1.46	0.0024

Biochemical parameters	Mean	SEM	Mean	SEM	*p* value
Total-cholesterol (mg/dl)	198.33	4.03	215.39	4.72	0.0068
HDL-cholesterol (mg/dl)	63.35	1.78	56.44	2.09	0.013
LDL-cholesterol (mg/dl)	114.4	3.46	131.54	4.05	0.0016

Biochemical parameters	Median	25th–75th Percentiles	Median	25th–75th Percentiles	*p* value
Triglycerides (mg/dl)	81	59–123	134	91–165	< 0.0001
HbA1c (%)	5.5	5.3–5.7	5.6	5.4–6	0.015
Insulin (uIU/ml)	3.6	2.9–5	4.9	3.5–8.5	0.001
Glucose (mg/dl)	81	75.5–86	83	79–90	0.047
HOMA-R	0.77	0.55–1.005	1.09	0.66–1.8	0.0007

Data were analyzed using unpaired *t*-test or Mann–Whitney *U* test to compare groups.

*NO* non-obese, *OB* obese.

When subjects were divided into MHO and MUO, various significant differences remained (Table 2). Specifically, MHO and MUO showed differences in SBP (MHO 133.61 ± 2.31, MUO 142.76 ± 3.71, *p* = 0.04) and DBP (MHO 78.69 ± 1.34, MUO 85.9 ± 2.14, *p* = 0.0056), which were higher in the MUO. A number of biochemical parameters were also elevated in the MUO compared to the MHO, with the MUO revealing significantly higher levels of TC (MHO 208.06 ± 4.97, MUO 227.14 ± 7.97, *p* = 0.046), HDL-C (MHO 59.76 ± 1.95, MUO 51.33 ± 3.12, *p* = 0.025), TG (MHO 94 (70.75–146), MUO 176 (159–202.5), *p* < 0.0001), insulin (MHO 4.2 (2.875–6.95), MUO 7.3 (3.9–10), *p* = 0.0047), glucose (MHO 83 (78–89.25), MUO 88 (80–95), *p* = 0.074) and HOMA-R (MHO 0.89 (0.59–1.42), MUO 1.66 (0.78–2.25), *p* = 0.0044), respectively.

**Table 2:** Characteristics of MHO and MUO.

Anthropometric and clinical parameters	MHO		MUO		*p* value
	Mean	SEM	Mean	SEM	
n (M:F)	54 (33:21)		21 (12:9)		
Age (years)	57	1.62	55	2.61	0.42
Height (cm)	161.68	1.13	161.11	1.81	0.79
Body weight (kg)	68.45	1.49	72.36	2.4	0.17
BMI (kg/m^2^)	26.16	0.46	27.77	0.73	0.066
Waist circumference (cm)	94.07	1.02	97.1	1.63	0.12
Systolic blood pressure (mmHg)	133.61	2.31	142.76	3.71	0.04
Diastolic blood pressure (mmHg)	78.69	1.34	85.9	2.14	0.0056

Biochemical parameters	Mean	SEM	Mean	SEM	*p* value
Total-cholesterol (mg/dl)	208.06	4.97	227.14	7.97	0.046
HDL-cholesterol (mg/dl)	59.76	1.95	51.33	3.12	0.025
LDL-cholesterol (mg/dl)	126.02	4.09	137.13	6.56	0.15

Biochemical parameters	Median	25th–75th Percentiles	Median	25th–75th Percentiles	*p* value
Triglycerides (mg/dl)	94	70.75–146	176	159–202.5	< 0.0001
HbA1c (%)	5.5	5.3–5.7	5.8	5.45–6	0.056
Insulin (uIU/ml)	4.2	2.875–6.95	7.3	3.9–10	0.0047
Glucose (mg/dl)	83	78–89.25	88	80–95	0.074
HOMA-R	0.89	0.59–1.42	1.66	0.78–2.25	0.0044

Data were analyzed using unpaired *t*-test or Mann–Whitney *U* test to compare groups.

*MHO* metabolic healthy obese, *MUO* metabolic unhealthy obese.

### Gut microbiota analysis

**Figure 2:**
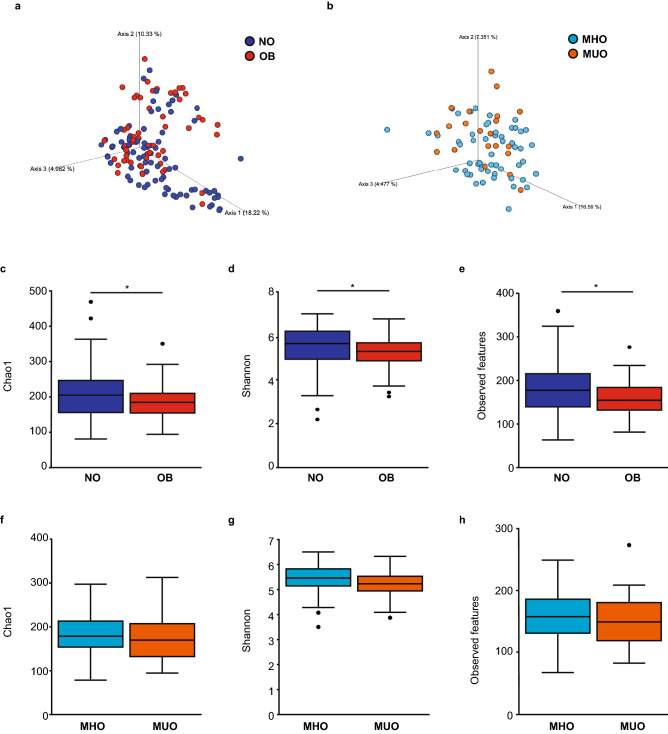
Diversity of gut microbiota in study subjects. Principal component analysis plot of microbiota based on unweighted UniFrac distances showing beta diversity in (**a**) NO and OB, and (**b**) MHO and MUO with permutational multivariate analysis of variance (PERMANOVA) employed to compare differences between groups. Alpha diversity was measured using Chao1, Shannon and Observed features indices to compare NO and OB (**c–e**), and MHO and MUO (**f–h**), with the Kruskal–Wallis test used to calculate the level of significance between groups. NO; non-obese (blue) (n = 81), OB; obese (red) (n = 59), MHO; metabolically healthy obese (light blue) (n = 54) and MUO; metabolically unhealthy obese (orange) (n = 21). Graphics were made using the QIIME2 (version: 2019.7). *p < 0.05.

A number of significant differences were observed between the gut microbiota communities of NO and OB as well as MHO and MUO. Firstly, beta diversity (dissimilarity index), as demonstrated by Principal Coordinates Analysis (PCoA), with percentage of variation described by Axis 1 (18.22%), Axis 2 (10.33%) and Axis 3 (4.982%) with the visual observation confirmed by the PERMANOVA test, which produced significant results for the comparison of NO and OB (*p* = 0.003) (Fig. 2a). When comparing beta diversity between MHO and MUO, PCoA showed variation described by Axis 1 (16.59%), Axis 2 (7.351%) and Axis 3 (4.477%), and confirmed by the PERMANOVA test, which did not produce significant results for the comparison of MHO and MUO (*p* = 0.077) (Fig. 2b). Alpha diversity as assessed by the Chao1, Shannon and Observed features with the Kruskal–Wallis test was significantly higher in NO compared to OB (Chao1 (*p* = 0.033), Shannon (*p* = 0.017) and Observed feature (*p* = 0.024)) (Fig. 2c–e). An elevation in alpha diversity was also observed in MHO compared to MUO however this did not reach statistical significance (Chao1 (*p* = 0.6), Shannon (*p* = 0.18) and Observed feature (*p* = 0.85)) (Fig. 2f–h).

**Figure 3:**
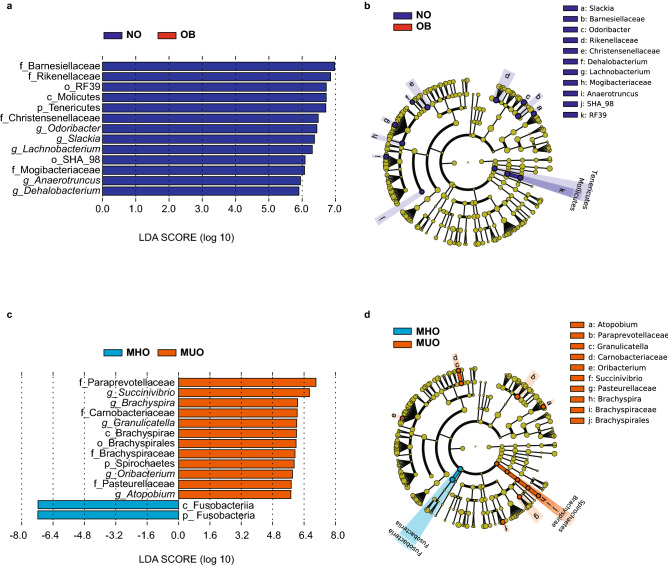
Linear discriminant analysis (LDA) score distribution of microbiome between (**a**) NO; non-obese (blue) and OB; obese (red), and (**c**) MHO; metabolically healthy obese (light blue) and MUO; metabolically unhealthy obese (orange). Alphabetical letters listed before the name of the enterobacteria represent p_; phylum, c_; class, o_; order, f_; family, and g_; genus levels. Linear discriminant analysis effect size (LEfSe) analyses of gut microbiota and Cladogram indicating statistical differences of the microbial populations between (**b**) NO; non-obese (blue) and OB; obese (red), and (**d**) MHO; metabolically healthy obese (light blue) and MUO; metabolically unhealthy obese (orange). Cladograms show the phylogenetic distribution of bacterial lineages associated with groups from kingdom, phylum, class, order, family, and genus levels are listed in kingdom from inside to outside of the cladogram. NO; non-obese (blue) (n = 81), OB; obese (red) (n = 59), MHO; metabolically healthy obese (light blue) (n = 54) and MUO; metabolically unhealthy obese (orange) (n = 21). Graphics were made using the Galaxy/Hutlab (https://huttenhower.sph.harvard.edu/galaxy/). *p* < 0.05, LDA score > 5.5.

Importantly, however, when employing the use of Linear Discriminant Analysis Effect Size (LEfSe) analyses, a range of marked differences in bacteria were observed at a variety of taxonomic levels between NO and OB as well as MHO and MUO. Notably, LEfSe analyses (*p* < 0.05, LDA score > 5.5) revealed that the ratio of NO versus OB had significantly higher levels in p_Tenericutes (*p* = 0.03, LDA score = 6.71) at the phylum level, in c_Molicutes (*p* = 0.03, LDA score = 6.72) at the class level and in o_SHA_98 (*p* = 0.049, LDA score = 6.09) and in o_RF39 (*p* = 0.03, LDA score = 6.73) at the order level, respectively. Abundance of microbiota including f_Barnesiellaceae (*p* = 0.02, LDA score = 6.98), f_Rikenellaceae (*p* = 0.007, LDA score = 6.86), f_Christensenellaceae (*p* = 0.034, LDA score = 6.48) and f_Mogibacteriaceae (*p* = 0.028, LDA score = 6.07) were elevated in NO compared to OB at the family level. The following genera were elevated in NO versus OB; *g_Slackia* (*p* = 0.023, LDA score = 6.37), *g_Odoribacter* (*p* = 0.04, LDA score = 6.44), *g_Dehalobacterium* (*p* = 0.004, LDA score = 5.9), *g_Lachnobacterium* (*p* = 0.03, LDA score = 6.31), *g_Anaerotruncus* (*p* = 0.049, LDA score = 5.95) (Fig. 3a, b).

In comparison, several kinds of bacteria at the phylum, class, order, family, and genus level were elevated in MUO as compared to MHO. At the phylum and class level, these included p_Spirochaetes (*p* = 0.022, LDA score = 5.9) and c_Brachyspirae (*p* = 0.022, LDA score = 6.01), respectively. Microbiota from the order o_Brachyspirales (*p* = 0.02, LDA score = 5.99) were elevated in MUO in comparison to MHO. At the family level, f_Paraprevotellaceae (*p* = 0.005, LDA score = 7.01), f_Pasteurellaceae (*p* = 0.02, LDA score = 5.75), f_Brachyspiraceae (*p* = 0.02, LDA score = 5.94) and f_Carnobacteriaceae (*p* = 0.03, LDA score = 6.05), were elevated in MUO compared to MHO, respectively. At the genus level, *g_Atopobium* (*p* = 0.0002, LDA score = 5.73), *g_Granulicatella* (*p* = 0.029, LDA score = 6.03), *g_Oribacterium* (*p* = 0.022, LDA score = 5.81)*, g_Succinivibrio* (*p* = 0.037, LDA score = 6.68)*, g_Brachyspira* (*p* = 0.022, LDA score = 6.08) were higher in MUO compared to MHO, respectively. Conversely, p_Fusobacteria (*p* = 0.039, LDA score = 7.17) and c_Fusobacteriia (*p* = 0.039, LDA score = 7.17) increased in MHO compared to MUO (Fig. 3c, d).

### Correlation of specific genera with components of the metabolic syndrome

**Figure 4:**
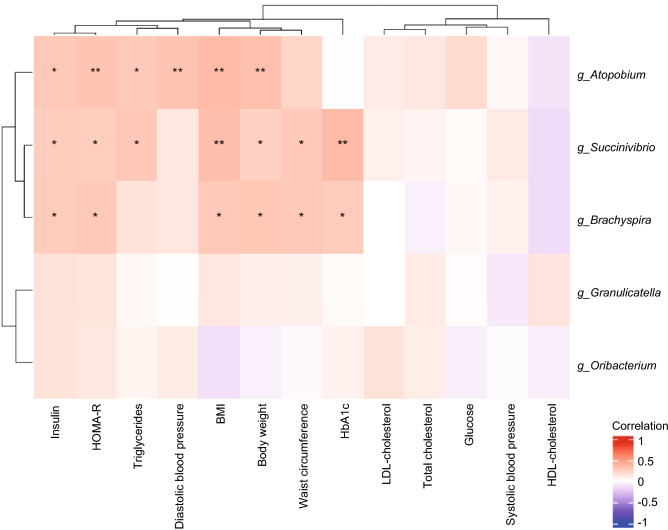
Spearman’s correlation heat map showing significant correlations of gut microbiota at the genus level and selected parameters in subjects with abdominal obesity (n = 75). Significant correlations are shown as red (positive) or blue (negative). Graphics were made using the R-package (version 4.0.3). *p < 0.05; **p < 0.01.

Regarding the relationship between various anthropometric, clinical, and biochemical parameters associated with metabolic syndrome (body weight, BMI, waist circumference, SBP, DBP, TC, HDL-C, LDL-C, TG, glucose, HbA1c, insulin and HOMA-R) and the five genera (*g_Atopobium*, *g_Granulicatella*, *g_Oribacterium, g_Succinivibrio, g_Brachyspira*), significant differences between MHO and MUO were shown in a heat map using Spearman's rank correlation coefficient (Fig. 4). Significant correlations were observed among three genera and the eight various anthropometric, clinical, and biochemical parameters associated with metabolic syndrome in the present study. In particular, *g_Atopobium* was positively associated with the value of Insulin (ρ = 0.28, *p* = 0.014), HOMA-R (ρ = 0.3, *p* = 0.009), TG (ρ = 0.27, *p* = 0.018), DBP (ρ = 0.3, *p* = 0.009), BMI (ρ = 0.35, *p* = 0.002) and body weight (ρ = 0.33, *p* = 0.004) , respectively, The genus *g_Succinivibrio* was positively correlated with the value of insulin (ρ = 0.26, *p* = 0.023), HOMA-R (ρ = 0.25, *p* = 0.028), TG (ρ = 0.28, *p* = 0.013), BMI (ρ = 0.33, *p* = 0.004), body weight (ρ = 0.23, *p* = 0.044), waist circumference (ρ = 0.28, *p* = 0.016) and HbA1c (ρ = 0.34, *p* = 0.002), respectively. Furthermore, *g_Brachtspira* was positively correlated with the value of insulin (ρ = 0.27, *p* = 0.02), HOMA-R (ρ = 0.28, *p* = 0.016), BMI (ρ = 0.28, *p* = 0.015), body weight (ρ = 0.28, *p* = 0.013), waist circumference (ρ = 0.27, *p* = 0.017) and HbA1c (ρ = 0.27, *p* = 0.021), respectively.

### Metabolite and SCFAs

**Figure 5:**
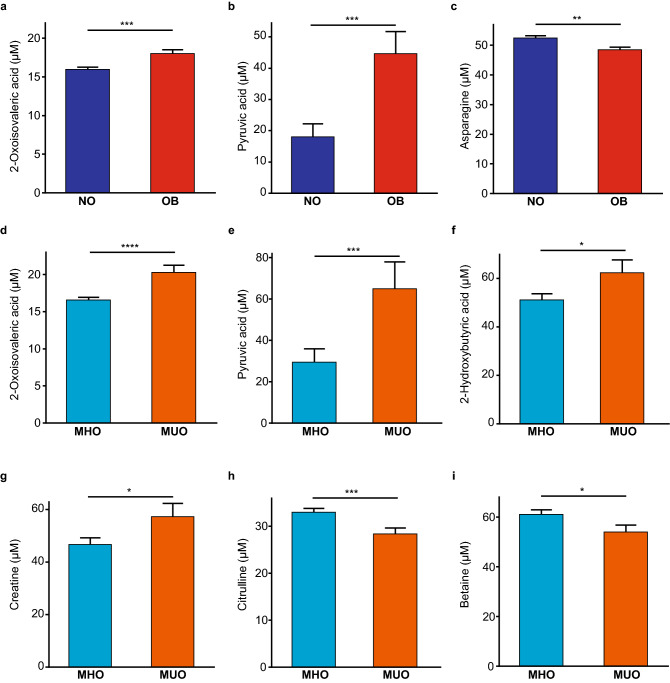
**(a–c**) Composition of selected metabolites in serum of NO and OB. (**d–i)** Composition of metabolites in serum of MHO and MUO. NO; non-obese (blue) (n = 81), OB; obese (red) (n = 59), MHO; metabolically healthy obese (light blue) (n = 54) and MUO; metabolically unhealthy obese (orange) (n = 21). Data are shown as mean ± SEM. Data were analyzed using unpaired *t*-test to compare groups. *p < 0.05; **p < 0.01; ***p < 0.001; ****p < 0.0001.

The concentration of SCFAs; acetate, propionate, butyrate and valerate, showed no significant differences between NO and OB as well as MHO and MUO (Supplementary Figs. S1 and S2). However, a number of significant differences were observed for other serum metabolites between NO and OB as well as MHO and MUO (Fig. 5). These included, 2-oxoisovaleric acid and pyruvic acid which were significantly elevated in OB versus NO (NO 15.96 ± 0.37, OB 18.01 ± 0.43, *p* = 0.0005 and NO 18.0 ± 5.07, OB 44.61 ± 5.94, *p* = 0.0009, respectively) (Fig. 5a, b). Whilst Asparagine was lower in OB compared to NO (NO 52.4 ± 0.82, OB 48.44 ± 0.97, *p* = 0.0022) (Fig. 5c). Between MHO and MUO, significant differences were observed for; 2-oxoisovaleric acid (MHO 16.55 ± 0.45, MUO 20.26 ± 0.73, *p* < 0.0001), pyruvic acid (MHO 29.39 ± 7.0, MUO 64.89 ± 11.22, *p* = 0.009), 2-hydroxybutyric acid (MHO 51.11 ± 2.85, MUO 62.35 ± 4.57, *p* = 0.04), and creatine (MHO 46.62 ± 2.76, MUO 57.21 ± 4.43, *p* = 0.046) (Fig. 5d–g) which were higher in MUO. In comparison, MHO compared to MUO showed higher levels of citrulline (MHO 32.97 ± 0.85, MUO 28.36 ± 1.36, *p* = 0.0051) and betaine (MHO 60.96 ± 1.86, MUO 53.88 ± 2.98, *p* = 0.047) (Fig. 5h, i).

## Discussion

The present study demonstrates that NO versus OB residents of Kumejima island, Okinawa, Japan showed distinctly different composition and diversity in gut microbiota. When subjects with abdominal obesity were divided into MHO and MUO, unique differences in the community of gut microbiota between the two groups were identified that correlated with aspects of the metabolic syndrome. Furthermore, MHO and MUO showed considerable differences in a series of serum metabolites that closely correlated with gut microbiota genera implicated in the pathophysiology of obesity.

Regarding the gut microbiota analyses, beta diversity was significantly different in NO compared to OB and alpha diversity, as measured by Chao1, Shannon and Observed features were significantly lower in OB as compared to NO. This finding is in line with majority of the literature, reproducing a consistent relationship between a lower alpha diversity and obesity phenotype^7,16^ whilst higher diversity is associated with stability and health of gut microbiota. It should be noted that measures of alpha and beta diversity were not significantly different between MHO and MUO in the present study, suggesting that such a relationship between microbial diversity and body weight would be stronger than other aspects of metabolic health including value of circulating lipids or HbA1c.

Furthermore, composition of gut microbiota showed a number of disparities between NO and OB as well as MHO and MUO. In particular, bacteria from the order SHA_98, which have been negatively correlated with BMI^17^, as well as RF39, which are correlated with reduced visceral fat and intestinal glucose intake^18^, were predominantly increased in NO compared to OB. Moreover, a series of bacteria which are characteristically reduced in obesity and obesity-related metabolic diseases including those from the Barnesiellaceae, Rikenellaceae and Christensenellaceae families^9,19,20^, were significantly elevated in NO compared to OB. Furthermore, bacteria from the Mogibacteriaceae family which are reported to be abundant in the intestinal bacteria of lean Japanese^21^, were significantly increased in NO as compared to OB. At the genus level, a number of genera were elevated in NO compared to OB including *Dehalobacterium* and *Odoribacter,* which have been shown to be reduced in the obese phenotype^9^, as well as *Slackia*, which have been implicated in host lipid and xenobiotic metabolism^22^, and *Anaeroturuncus*, which has been found to negatively correlate with BMI^23^. Also, bacteria from the genus *Lachnobacterium* were increased in NO compared to OB, which has been shown to have a negative correlation with body fat, LDL-C and TC^24^.

Moreover, when subjects were divided into MHO and MUO, a number of significant differences in the gut microbiota were identified. Notably, bacteria from the family Paraprevotellaceae, which were elevated in MUO compared to MHO, have been shown in previous studies to be positively correlated with circulating blood TG, TC, and glucose tolerance^25^. Regarding differences between groups at the genus level, *Atopobium* has been known to increase under conditions of hormonal and metabolic dysfunction including polycystic ovary syndrome (PCOS). To extend on this, the Coriobacteriaceae family, to which *Atopobium* belong, have been associated with increased risk of the metabolic syndrome^26^. Following on from this, Spearman’s correlation analyses in the present study support a line of previous findings, revealing that *Atopobium* showed a significant positive correlation with plasma levels of fasting insulin, HOMA-R, TG and Diastolic blood pressure, in addition to BMI and body weight. These results suggest that increased *Atopobium* would play a role in the pathophysiology of MUO.

Among a line of other genera significantly increased in MUO in the present study, *Granulicatella* is known to decrease in the oral cavity of obese patients after the metabolic surgery^27^, and *Oribacterium* has been reported as a potential obesogenic bacteria^28^. On the other hand, *Succinivibrio*, a bacterium known to correlate with risk of type 2 diabetes and obesity disease^29^ was significantly correlated with plasma fasting insulin, HOMA-R, TG, BMI, weight, waist circumference, and HbA1c in present study. Furthermore, *Brachyspira* is known to colonize in the intestinal mucosa, implicating in the pathogenesis of irritable bowel syndrome^30^. Conversely, the only gut microbiota that increased in MHO compared MUO were bacteria from the class Fusobacteriia, phylum Fusobacteria. Fusobacteriia are butyrate-producing bacteria^31^, suggesting that elevated levels of this bacteria may result in preserved metabolic health among obese individuals.

Pertaining to analyses of gut microbiota associated serum metabolites, we did not observe any significant differences in the concentration of SCFAs among the groups. However, we did observe a number of notable differences in the levels of specific serum metabolites implicated in energy homeostasis and glucose metabolism between NO and OB as well as MUO and MHO. Of note, we found that 2-oxoisovaleric acid and pyruvic acid were significantly elevated in OB and MUO in comparison to NO and MHO. In line with our data, 2-oxoisovaleric acid is implicated in mitochondrial dysfunction and elevated in subjects with obesity and T2DM^32,33^. Furthermore, higher levels of pyruvic acid are known to promote gluconeogenesis and also commonly observed in patients with T2DM^34^. On the other hand, we found that level of serum asparagine was significantly reduced in OB in comparison to NO. In fact, serum level of asparagine has been shown to increase after weight loss in humans^35^ as well as to associate with adaptive thermogenesis via mTORC1 signaling in mice^36^. Therefore, elevation in serum level of asparagine would facilitate heat production, thereby contributing to maintenance of body shape. In addition, level of creatine in serum, which was significantly elevated in MUO compared to MHO in the present study, has been shown to positively associate with certain pathogenic bacteria and dysbiosis in a mouse model of acute kidney injury, which often ensues with metabolic syndrome^37^. Furthermore, 2-hydroxybutyric acid, which was significantly elevated in serum from MUO versus MHO in the present study, has been shown to be implicated in oxidative stress, thereby highlighting its usefulness as an early biomarker for insulin resistance^38^. In contrast, citrulline, which was significantly reduced in serum of MUO compared to MHO in the present study, appears to play a role in arterial dilation and glucose homeostasis^39^. A randomized double-blind-placebo controlled study revealed that citrulline supplementation was able to markedly reduce value of fasting blood glucose and HbA1c in humans^40^. Lastly, concentration of betaine in serum, a naturally occurring methyl donor, which was significantly decreased in the MUO compared to MHO, is known to associate with metabolically-beneficial profile in Asian men with mild fatty liver disease^41^. The possible relationships between four kinds of serum metabolites (2-oxoisovaleric acid, pyruvic acid, 2-hydroxybutyric acid, and creatine) and the five kinds of genera (*g_Atopobium, g_Granulicatella, g_Oribacterium, g_Succinivibrio, g_Brachyspira*) showing significant differences between MHO and MUO were explored. However, overall, the strength of the correlation (ρ) among respective parameters was extremely subtle, yielding no definite links worth consideration (Supplementary Fig. S3).

We do recognize that there are a couple of limitations in the present study. Firstly, the cross-sectional nature limits the ability to make causal inferences regarding the relationship between specific microbiota and serum metabolites with obesity and markers of metabolic health. Second, abdominal obesity in the present study was classified according to the Japanese standard^42^, with waist circumference measured at the umbilical level and therefore generalizability to other population groups needs to be cautioned. However, results obtained are valuable when taking other Asian populations into consideration who commonly show signs of metabolic dysfunction at a relatively low BMI. Although the size of the sample may present another limitation, it should be noted that overall lifestyle including daily food consumption and physical activities in the remote island show a similar trend among inhabitants, which may be advantageous to obtain solid data on the profile of gut microbiota and microbial metabolites given the relatively small population size^43^.

In conclusion, our data demonstrate that stark differences in the gut microbiota and metabolite profile exist between not only obese and healthy weight individuals but also between MHO and MUO inhabitants of the remote island, where the prevalence of obesity is one of the highest in Japan. Overall, our data may help to pave a new avenue in approaching the multidimensional nature of obesity and the need for a tailored approach for the treatment of MHO and MUO that better focuses on the unique profile of gut microbiota and serum metabolites.

## Methods

### Study design and population

This cross-sectional study targeted residents in Kumejima Island, Okinawa prefecture, in Japan aged over 20 years as a part of the KDHP (Kumejima Digital Health Project) between June 2018 and December 2019. KDHP is a Japanese national project designed to observe the health status of residents in Kumejima Island over time and with a particular emphasis on the profile of gut microbiota and serum metabolites, to characterize lifestyle-related diseases, including obesity and T2DM which this population group is vulnerable to. Participants were residents in Kumejima island over 20 years old, except for those met the following exclusion criteria; (1) severe renal dysfunction (eGFR < 30 ml/min/1.73 m^2^) or hepatic dysfunction (AST > 200 IU/L or ALT > 200 IU/L), (2) malignancy, (3) pregnancy, (4) chronic diarrhea, or (5) severe illness requiring hospitalization and treatment. A total of 178 subjects were recruited with 140 individuals (70 females and 70 males) able to provide all the required measurements and samples including anthropometric measurements, clinical and biochemical blood samples as well as stool samples.

Initially, all subjects were classified into NO and OB defined as a BMI < 25 and ≥ 25 kg/m^2^^2^, respectively, to compare the profiles of interest. Next, with reference to the Japanese definition of the metabolic syndrome^42^, subjects with abdominal obesity defined as a waist circumference ≥ 85 cm for men and ≥ 90 cm for women were further divided into MHO and MUO. Specifically, subjects were classed as MHO if they had no apparent metabolic disorders, whilst MUO subjects were classified as having at least two of the following three metabolic abnormalities; high blood glucose, dyslipidemia, and high blood pressure. High blood glucose was defined as a fasting blood glucose ≥ 100 mg/dL or use of medication for T2DM, dyslipidemia defined as a fasting serum TG ≥ 150 mg/dL or HDL-C < 40 mg/dL, high blood pressure defined as SBP ≥ 130 mmHg, DBP ≥ 85 mmHg, or use of medication for hypertension^42^ (Fig. 1). All participants provided written informed consent and the study was approved by the Ethics Committee of University of the Ryukyus for Medical and Health Research Involving Human Subjects (No.1194) in accordance with the Declaration of Helsinki.

### Sample and basic data collection

Participant’s fasting blood samples were drawn from an ulnar median cutaneous vein using a 20 ml disposable syringe (Nipro Medical, Osaka, Japan) with a 23 G butterfly needle (Nipro Medical, Osaka, Japan) and transferred to a blood-collection tube (NP-FN0205, Nipro Neo-tube; Nipro Medical, Osaka, Japan). Circulating TC was measured by Determiner C-TC (Minaris Medical Co., Ltd., Tokyo, Japan), circulating HDL-C was measured by Metabolead HDL-C (Minaris Medical Co., Ltd., Tokyo, Japan) and circulating LDL-C calculated by Friedewald^44^. Value of circulating TG was measured by Determiner C-TG (Minaris Medical Co., Ltd., Tokyo, Japan). Value of HbA1c in blood was analyzed with Tosoh Automated Glycohemoglobin Analyzer HLC-723G8 (TOSOH Corporation, Shiga, Japan). Plasma insulin concentrations were measured by ARCHITECT Insulin (Abbott Japan LLC, Tokyo, Japan). Plasma glucose was measured by l-type Wako Glu2 (FUJIFILM Wako Pure Chemical Corporation, Osaka, Japan). HOMA-R calculations were performed according to the following formula: (concentration of fasting blood glucose (mmol/l)) × (concentration of fasting blood insulin (mU/l))/22.5^45^. Blood pressure and anthropometric indices were measured by trained nurses using standard manners. Within 0–7 days of blood collection, participants provided fecal samples, using a fecal collection container No. 4 with cap, P-sagittate, with round label type 2 affixed (ASIAKIZAI Co., Tokyo, Japan).

### Metabolite analysis

Fasting serum samples were collected in 1.5 ml micro centrifuge tubes (CF-0150, BIO-BIK; Ina Optika, Osaka, Japan) and stored at − 80 °C until processing. Human serum was analyzed using capillary electrophoresis time-of-flight mass spectrometry (CE-TOFMS); (Agilent Technologies, CA, USA) at Human Metabolome Technology Inc. (HMT) (Human Metabolome Technologies, Yamagata, Japan). For measurements using CE-TOFMS, 200 µl of methanol solution adjusted to concentration of 20 µl of internal standards (Human Metabolome Technologies, Yamagata, Japan), 50 µl of human serum was added and stirred. To this was added 150 µl of Milli-Q water, stirred, and 300 µl transferred to an ultrafiltration tube (Centrifugal Filter Unit 5 kDa; Ultra-free MC PLHCC, Human Metabolome Technologies, Yamagata, Japan), and centrifuged (9,100 × g, 4 °C, 120 min) and subjected to ultrafiltration. The filtrate was allowed to dry, dissolved again in 50 µl of Milli-Q water for Capillary electrophoresis is coupled to mass spectrometry (CE-MS) analysis. CE-TOFMS was used to measure cation and anion mode under the conditions shown in Supplementary Table S1^46–48^. Judging from the peak intensities and shapes obtained, the dilution factors were standardized to cation:1, anion:5, for all samples. The detected peaks were automatically extracted using the automatic integration software MasterHands (ver. 2.17.1.11; Keio University, Tokyo, Japan) for peaks with a signal-to-noise (S/N) ratio of 3 or higher, and the mass-to-charge ratio (m/z), peak area value, and Migration time (MT) in CE-TOFMS. The obtained peak area values were converted to relative area values using the following equation (Relative area value = Area ratio of target peak/Area value of internal RMs*Sample volume). Since these data contain adduct ions such as Na^+^ and K^+^ and fragment ions such as dehydration and diammonium, these molecular weight-related ions were removed. However, since there were also substance-specific adducts and fragments, it was not possible to scrutinize all of them. Peaks that were scrutinized were matched and aligned between each sample based on m/z and MT values. The detected peaks were matched and searched against all substances registered in the HMT Metabolite Library and the Known-Unknown Library based on m/z and MT values. Tolerances for searching candidate metabolites in serum were ± 0.5 min for MT and ± 10 ppm (CE-TOFMS) for m/z.

### SCFAs analysis

Concentration of SCFAs (acetic acid, propionic acid, butyric acid and valeric acid) in from subjects’ serum were measured by Liquid chromatography-tandem mass spectrometry (LC–MS/MS). The serum samples and the internal standard solution (40 µmol/L; Acetic acid-d4) were deproteinized with methanol. For the calibration curves, standard solutions of SCFAs (Merck, Darmstadt, Germany) were spiked into the surrogate matrix for the ultrapure water instead of serum to prepared calibration curves. These samples were derivatization with 2-nitrophenyl hydrazine and each of samples were extracted by using t-butyl methyl ether.

After the solvent was evaporated to dryness under a stream of nitrogen gas, the residue was dissolved in final solvent. The processed sample was injected into the LC–MS/MS system. Regarding the LC–MS/MS system, liquid chromatography was performed using an ACQUITY UPLC system (Waters Corporation, Massachusetts, USA), separated using an analytical column (AQUITY BEH C18, 2.1 × 100 mm, 1.7 μm; Waters Corporation, Massachusetts, US). Electrospray ionization (ESI) was carried out with the API4000, Tripre Quad 500 and QTRAP 5500 (Sciex, Massachusetts, USA) operating in the negative ionization and SRM mode. The SRM transitions for acetic acid, propionic acid, butyric acid and valeric acid were m/z 194–164, m/z 208–178, m/z 222–192 and 236–206 respectively.

### DNA extraction and 16S rRNA sequencing

Fecal samples were stored at -80 °C until processing and then genomic DNA was extracted using the NucleoSpin Microbial DNA (Macherey–Nagel, Duren, Germany) according to the manufacturer’s instructions. All extracted DNA samples were quantified by fluorescence using Quant-iT dsDNA Assay Kit (Thermo Fisher Scientific, Massachusetts, USA) and purified using the Agencourt AMPure XP (Beckman Coulter, CA, USA). Sequencing libraries were prepared using the 16S (V3-V4) Metagenomic Library Construction Kit for NGS (Takara Bio, Kusatsu, Japan). The first PCR amplification was performed using the primer pair 341 F (5’-TCGTCGGCAGCGTCAGATGTGTATAAGAGACAGCCTACGGGNGGCWGCAG-3’) and 806 R (5’-GTCTCGTGGGCTCGGAGATGTGTATAAGAGACAGGGACTACHVGGGTWTCTAAT-3’) with Illumina adaptor overhang sequences. The second PCR amplification was performed using the Nextera XT Index Kit v2 (Illumina, San Diego, USA). Sequencing libraries were purified using the Agencourt AMPure XP (Beckman Coulter) and quantified by fluorescence using the Quant-iT dsDNA Assay Kit (Thermo Fisher Scientific). Clonal clusters of the libraries were generated and sequenced on a MiSeq system (Illumina) with the MiSeq Reagent v3 kit in 2 × 250 bp mode.

Raw sequences were filtered using QIIME2 (version:2019.7)^49^ and were demultiplexed by per-sample barcodes and Illumina-sequenced amplicon read errors were corrected by DADA2 and clustered into operational taxonomic units (OTU) at 99% identity using the VSEARCH. Taxonomy was classified using the GreenGenes 99% OTUs database (version 13_8). A rarefaction curve was generated after random sampling up to 50,000 sequences in each sample. Alpha diversity metrics were calculated for each sample at the sampling depth. To test the association for each taxonomy and species, low abundance species were first filtered and then analyses were conducted using 369 OTUs (shared in more than 50% of samples).

### Statistical analysis

Data were shown as mean ± standard error of the mean (SEM) for normal distribution variables, and as median and 25th–75th percentiles for skewed distribution variables. Comparisons between NO and OB, as well as between MHO and MUO, for serum TG, HbA1c, insulin, glucose and HOMA-R were performed using Mann–Whitney *U* test with unpaired *t*-test used for the other parameters. Both alpha and beta diversities were calculated using QIIME2 (version: 2019.7)^49^. Unweighted UniFrac distance metrics were obtained to generate principal coordinate analysis (PCoA). The community structure between NO and OB, and MHO and MUO, was compared by measuring Chao1, Shannon and Observed features. Statistical differences between NO and OB, as well as MHO and MUO at different taxonomic assignments were calculated using Linear Discriminant Analysis Effect Size (LEfSe) using criteria, *p* < 0.05, LDA score > 5.5. LEfSe were performed Galaxy Huttenhower Lab platform^50^. Comparisons of metabolites and SCFAs between NO and OB, and MHO and MUO groups were analyzed using unpaired *t*-test, and correlation analysis of metabolites and gut microbiota was analyzed using Spearman’s rank correlation analysis with JMP version 15.0.0. Spearman’s rank correlation analysis was used to examine the nature of associations between gut microbiota taxa and clinical indices as well as selected metabolites in the serum of subjects with abdominal obesity using the R-package (version 4.0.3), employing the *cor* function, *Circlize* and *ComplexHeatmap* from R-package to generate heat maps. Other statistical analyses were performed using JMP version 15.0.0 (SAS Institute Inc., Cary, NC, USA). Probability values were two-tailed, with levels of statistical significance set at *p* < 0.05.

## Supplementary Information

Supplementary Information.

## Data Availability

The datasets generated and/or analysed during the current study are available in Mendeley Data repository, https://data.mendeley.com/datasets/z483h65xrp/2

## References

[1] Bluher M (2019). Obesity: Global epidemiology and pathogenesis. Nat. Rev. Endocrinol..

[2] Shiwaku K (2004). Overweight Japanese with body mass indexes of 23.0–24.9 have higher risks for obesity-associated disorders: A comparison of Japanese and Mongolians. Int. J. Obes. Relat. Metab. Disord..

[3] Brunner E, Cable N, Iso H (2021). Health in Japan: Social Epidemiology of Japan Since the 1964 Tokyo Olympics.

[4] de la Cuesta-Zuluaga J, Corrales-Agudelo V, Carmona JA, Abad JM, Escobar JS (2018). Body size phenotypes comprehensively assess cardiometabolic risk and refine the association between obesity and gut microbiota. Int. J. Obes. (Lond.).

[5] Xu Z (2022). Gut microbiota in patients with obesity and metabolic disorders: A systematic review. Genes Nutr..

[6] Crovesy L, Masterson D, Rosado EL (2020). Profile of the gut microbiota of adults with obesity: A systematic review. Eur. J. Clin. Nutr..

[7] Stanislawski MA, Dabelea D, Lange LA, Wagner BD, Lozupone CA (2019). Gut microbiota phenotypes of obesity. NPJ Biofilms Microbiomes.

[8] Chen Z (2021). Association of insulin resistance and type 2 diabetes with gut microbial diversity: A microbiome-wide analysis from population studies. JAMA Netw. Open.

[9] Peters BA (2018). A taxonomic signature of obesity in a large study of American adults. Sci. Rep..

[10] Gill SK, Rossi M, Bajka B, Whelan K (2021). Dietary fibre in gastrointestinal health and disease. Nat. Rev. Gastroenterol. Hepatol..

[11] Sharma A (2019). Impaired skeletal muscle mitochondrial pyruvate uptake rewires glucose metabolism to drive whole-body leanness. Elife.

[12] Gupta A (2019). Association of *Flavonifractor plautii*, a flavonoid-degrading bacterium, with the gut microbiome of colorectal cancer patients in India. mSystems.

[13] Arsenault BJ, Beaumont EP, Despres JP, Larose E (2012). Mapping body fat distribution: A key step towards the identification of the vulnerable patient?. Ann. Med..

[14] Phillips CM, Perry IJ (2013). Does inflammation determine metabolic health status in obese and nonobese adults?. J. Clin. Endocrinol. Metab..

[15] Wildman RP (2008). The obese without cardiometabolic risk factor clustering and the normal weight with cardiometabolic risk factor clustering: Prevalence and correlates of 2 phenotypes among the US population (NHANES 1999–2004). Arch. Intern. Med..

[16] Ballini A, Scacco S, Boccellino M, Santacroce L, Arrigoni R (2020). Microbiota and obesity: Where are we now?. Biology (Basel).

[17] Ottosson F (2018). Connection between BMI-related plasma metabolite profile and gut microbiota. J. Clin. Endocrinol. Metab..

[18] Guzzardi MA (2021). Maturation of the visceral (gut-adipose-liver) network in response to the weaning reaction versus adult age and impact of maternal high-fat diet. Nutrients.

[19] Del Chierico F (2018). Gut microbiota markers in obese adolescent and adult patients: Age-dependent differential patterns. Front. Microbiol..

[20] Li R, Andreu-Sanchez S, Kuipers F, Fu J (2021). Gut microbiome and bile acids in obesity-related diseases. Best Pract. Res. Clin. Endocrinol. Metab..

[21] Oki K (2016). Comprehensive analysis of the fecal microbiota of healthy Japanese adults reveals a new bacterial lineage associated with a phenotype characterized by a high frequency of bowel movements and a lean body type. BMC Microbiol..

[22] Cho GS (2016). Quantification of Slackia and Eggerthella spp. in human feces and adhesion of representatives strains to Caco-2 cells. Front. Microbiol..

[23] Lv YR (2019). The association between gut microbiota composition and BMI in Chinese male college students, as analysed by next-generation sequencing. Br. J. Nutr..

[24] Companys J (2021). Gut microbiota profile and its association with clinical variables and dietary intake in overweight/obese and lean subjects: A cross-sectional study. Nutrients.

[25] Gao F (2019). Butyrate improves the metabolic disorder and gut microbiome dysbiosis in mice induced by a high-fat diet. Front. Pharmacol..

[26] Del Chierico F (2021). Fecal microbiota signatures of insulin resistance, inflammation, and metabolic syndrome in youth with obesity: A pilot study. Acta Diabetol..

[27] Dzunkova M (2020). Salivary microbiome composition changes after bariatric surgery. Sci. Rep..

[28] Angelakis E (2015). A metagenomic investigation of the duodenal microbiota reveals links with obesity. PLoS ONE.

[29] Miller TL, Wolin MJ (1979). Fermentations by saccharolytic intestinal bacteria. Am. J. Clin. Nutr..

[30] Jabbar KS (2021). Association between Brachyspira and irritable bowel syndrome with diarrhoea. Gut.

[31] Rajilic-Stojanovic M, de Vos WM (2014). The first 1000 cultured species of the human gastrointestinal microbiota. FEMS Microbiol. Rev..

[32] Concepcion J (2020). Identification of pathognomonic purine synthesis biomarkers by metabolomic profiling of adolescents with obesity and type 2 diabetes. PLoS ONE.

[33] Vogelzangs N (2020). Metabolic profiling of tissue-specific insulin resistance in human obesity: Results from the Diogenes study and the Maastricht Study. Int. J. Obes. (Lond.).

[34] Konrad T (1999). alpha-Lipoic acid treatment decreases serum lactate and pyruvate concentrations and improves glucose effectiveness in lean and obese patients with type 2 diabetes. Diabetes Care.

[35] Geidenstam N, Al-Majdoub M, Ekman M, Spegel P, Ridderstrale M (2017). Metabolite profiling of obese individuals before and after a one year weight loss program. Int. J. Obes. (Lond.).

[36] Xu Y (2021). Asparagine reinforces mTORC1 signaling to boost thermogenesis and glycolysis in adipose tissues. EMBO J..

[37] Andrianova NV (2020). Microbiome-metabolome signature of acute kidney injury. Metabolites.

[38] Sousa AP (2021). Which role plays 2-hydroxybutyric acid on insulin resistance?. Metabolites.

[39] Pi X, Xie L, Patterson C (2018). Emerging roles of vascular endothelium in metabolic homeostasis. Circ. Res..

[40] Abbaszadeh F, Azizi S, Mobasseri M, Ebrahimi-Mameghani M (2021). The effects of citrulline supplementation on meta-inflammation and insulin sensitivity in type 2 diabetes: A randomized, double-blind, placebo-controlled trial. Diabetol. Metab. Syndr..

[41] Tiihonen K, Saarinen MT (2016). Effect of dietary betaine on metabolic syndrome risk factors in Asian. J. Diabetes Metab..

[42] Definition and the diagnostic standard for metabolic syndrome—Committee to Evaluate Diagnostic Standards for Metabolic Syndrome. *Nihon Naika Gakkai Zasshi*. **94**, 794–809 (2005). 15865013

[43] Mobegi FM (2020). Intestinal microbiology shapes population health impacts of diet and lifestyle risk exposures in Torres Strait Islander communities. Elife.

[44] Friedewald WT, Levy RI, Fredrickson DS (1972). Estimation of the concentration of low-density lipoprotein cholesterol in plasma, without use of the preparative ultracentrifuge. Clin. Chem..

[45] Matthews DR (1985). Homeostasis model assessment: insulin resistance and beta-cell function from fasting plasma glucose and insulin concentrations in man. Diabetologia.

[46] Soga T, Heiger DN (2000). Amino acid analysis by capillary electrophoresis electrospray ionization mass spectrometry. Anal. Chem..

[47] Soga T (2002). Simultaneous determination of anionic intermediates for Bacillus subtilis metabolic pathways by capillary electrophoresis electrospray ionization mass spectrometry. Anal. Chem..

[48] Soga T (2003). Quantitative metabolome analysis using capillary electrophoresis mass spectrometry. J. Proteome Res..

[49] Bolyen E (2019). Reproducible, interactive, scalable and extensible microbiome data science using QIIME 2. Nat. Biotechnol..

[50] Segata N (2011). Metagenomic biomarker discovery and explanation. Genome Biol..

