# Supplementary material for: Profile of gut microbiota and serum metabolites associated with metabolic syndrome in a remote island most afflicted by obesity in Japan
Source: Sci Rep. 2022 Oct 14;12:17292. doi: 10.1038/s41598-022-21708-0 (PMC9568508; doi:10.1038/s41598-022-21708-0)
Supplement: Supplementary file 1 — Supplementary Information. [file 41598_2022_21708_MOESM1_ESM.pdf]

Profile of gut microbiota and serum metabolites associated with metabolic syndrome in a remote island most afflicted by obesity in Japan

Tsugumi Uema[1], Jasmine F. Millman[1*], Shiki Okamoto[1*], Takehiro Nakamura[1], Kiyoto Yamashiro[1], Moriyuki Uehara[1], Ken-ichiro Honma[1], Minoru Miyazato[2], Asuka Ashikari[3], Seiichi Saito[3], Shiro Maeda[4,5], Minako Imamura[4,5], Hajime Ishida[6], Masayuki Matsushita[7], Koshi Nakamura[8], Hiroaki Masuzaki[1*]

[1] Division of Endocrinology, Diabetes and Metabolism, Hematology, Rheumatology, (Second Department of Internal Medicine), Graduate School of Medicine, University of the Ryukyus, Okinawa, Japan

[2] Department of Systems Physiology, Graduate School of Medicine, University of the Ryukyus, Okinawa, Japan

[3] Department of Urology, Graduate School of Medicine, University of the Ryukyus, Okinawa, Japan

[4] Department of Advanced Genomic and Laboratory Medicine, Graduate School of Medicine, University of the Ryukyus, Okinawa, Japan

[5] Division of Clinical Laboratory and Blood Transfusion, University of the Ryukyus Hospital, Okinawa, Japan

[6] Department of Human Biology and Anatomy, Graduate School of Medicine, University of the Ryukyus, Okinawa, Japan

[7] Department of Molecular and Cellular Physiology, Graduate School of Medicine, University of the Ryukyus, Okinawa, Japan

[8] Department of Public Health and Hygiene, Graduate School of Medicine, University of the Ryukyus, Okinawa, Japan

*** Co-corresponding authors:**

Hiroaki Masuzaki

207 Uehara, Nishihara, Okinawa, 903-0215, Japan

hiroaki@med.u-ryukyu.ac.jp

Tel: +81-98-895-1146, Fax: +81-98-895-1415

ORCID: 0000-0002-2445-1047

Shiki Okamoto

207 Uehara, Nishihara, Okinawa, 903-0215, Japan

shiki@med.u-ryukyu.ac.jp

Tel: +81-98-895-1146, Fax: +81-98-895-1415

ORCID: 0000-0002-2802-0527

Jasmine Frances Millman

207 Uehara, Nishihara, Okinawa, 903-0215, Japan

Jasmine.f.millman@gmail.com

Tel: +81-98-895-1146, Fax: +81-98-895-1415

ORCID: 0000-0001-7049-3881

Supplementary figure legends

Figure S1 Concentration of plasma short chain fatty acids (SCFAs) in NO and OB subjects. **a-d.** SCFA concentrations of acetate, propionate, butyrate, and valerate. **e.** Total concentration of plasma SCFAs. NO; non-obese (blue) (n = 81) and OB; obese (red) (n = 59). Data are shown as median and 25th – 75th percentiles. Data were analyzed using Mann-Whitney *U* test to compare groups.

Figure S2 Concentration of plasma SCFAs in MHO and MUO subjects. **a-d.** SCFA concentrations of acetate, propionate, butyrate, and valerate. **e.** Total concentration of plasma SCFAs. MHO; metabolically healthy obese (light blue) (n = 54) and MUO; metabolically unhealthy obese (orange) (n = 21). Data are shown as median and 25th – 75th percentiles. Data were analyzed using Mann-Whitney *U* test to compare groups.

Figure S3 Correlations between gut microbiota and selected metabolites in subjects with abdominal obesity (n=75) showing Spearman's correlation between gut microbiome (x axis) and metabolites (y axis). Dots represent each individual subject.

Table S1 Measurement conditions of CE-TOFMS

Figure S1

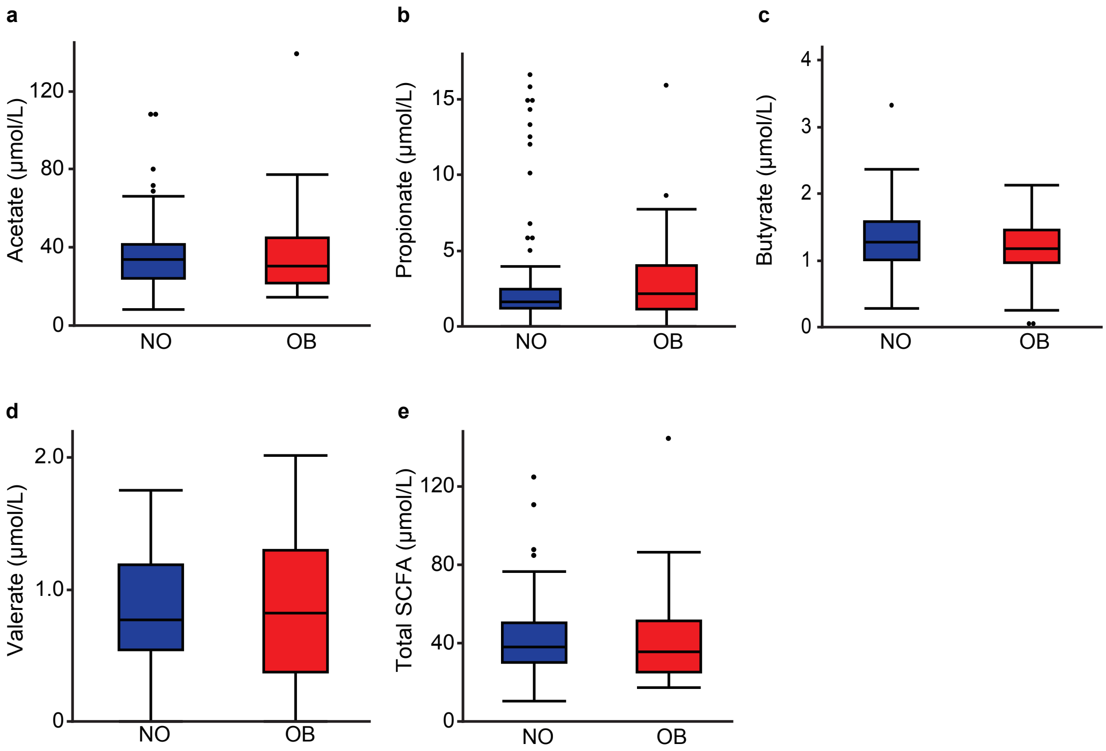
a
Acetate (µmol/L)
0
40
80
120
NO
OB
b
Propionate (µmol/L)
0
5
10
15
NO
OB
c
Butyrate (µmol/L)
0
1
2
3
4
NO
OB
d
Valerate (µmol/L)
0
1.0
2.0
NO
OB
e
Total SCFA (µmol/L)
0
40
80
120
NO
OB

Figure S2

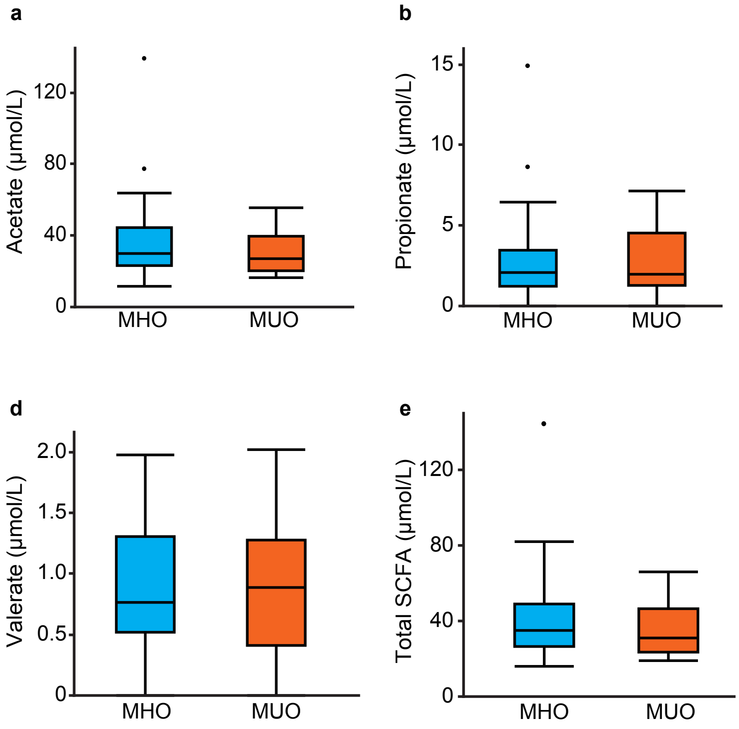
a
Acetate (μmol/L)
0
40
80
120
MHO
MUO
b
Propionate (μmol/L)
0
5
10
15
MHO
MUO
c
Butyrate (μmol/L)
0
0.5
1.0
1.5
2.0
MHO
MUO
d
Valerate (μmol/L)
0
0.5
1.0
1.5
2.0
MHO
MUO
e
Total SCFA (μmol/L)
0
40
80
120
MHO
MUO

Figure S3

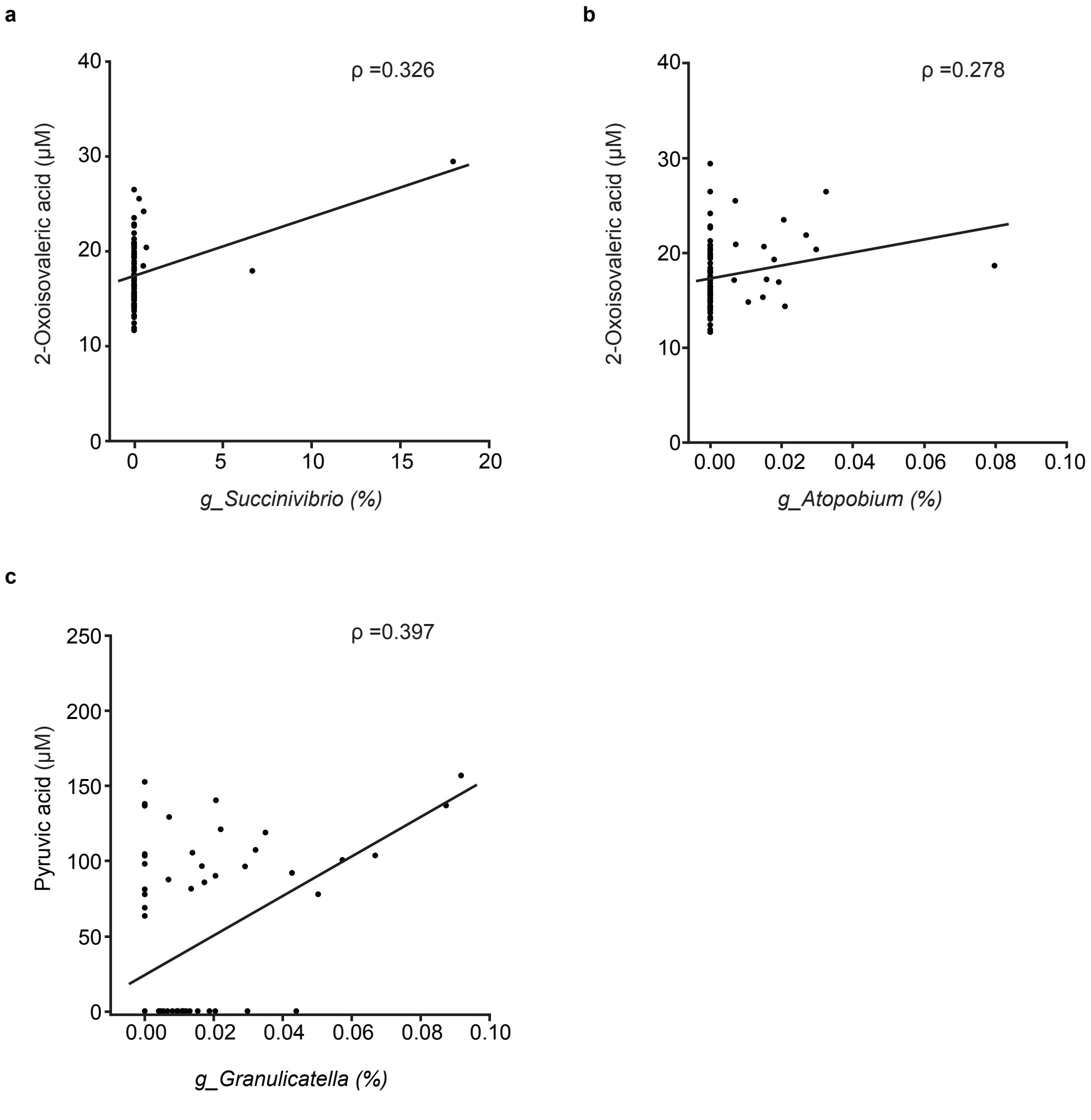
a
b
c
ρ =0.326
2-Oxoisovaleric acid (μM)
g_Succinivibrio (%)
ρ =0.278
2-Oxoisovaleric acid (μM)
g_Atopobium (%)
ρ =0.397
Pyruvic acid (μM)
g_Granulicatella (%)

Table S1; Mesuring conditions of CE-TOFMS

	cation	anion
Run buffer	Cation Buffer Solution (p/n :H3301-1001)	Ation Buffer Solution (p/n :I3302-1023)
Rinse buffer	Cation Buffer Solution (p/n :H3301-1001)	Ation Buffer Solution (p/n :I3302-1023)
Sample injection	Pressure injection 50 mbar, 5 sec	Pressure injection 50 mbar, 10 sec
CE voltage	Positive, 30kV	Positive, 30kV
MS ionization	ESI Positive	ESI Negative
MS capillary voltage	4,000V	3,500V
MS scan range	m/z 50-1,000	m/z 50-1,000
Sheath liquid	HMT Sheath Liquid (p/n :H3301-1020)	HMT Sheath Liquid (p/n :H3301-1020)
